# Experimental and empirical evidence shows that reducing weed control in winter cereal fields is a viable strategy for farmers

**DOI:** 10.1038/s41598-019-45315-8

**Published:** 2019-06-21

**Authors:** Rui Catarino, Sabrina Gaba, Vincent Bretagnolle

**Affiliations:** 1 CEBC, UMR 7372, CNRS & Université de la Rochelle, Villiers-en-Bois, F-79360 France; 2 USC 1339 Centre d’Etudes Biologiques de Chizé, INRA, F-76390 Villiers-en-Bois, France; 3 LTSER “Zone Atelier Plaine & Val de Sèvre”, CNRS, Villiers-en-Bois, F-79360 France

**Keywords:** Agroecology, Agroecology

## Abstract

Modern agriculture needs a paradigm shift to make the world’s food production sustainable while mitigating social and environmental externalities. Although various policies to limit the use of agrochemicals have recently been implemented in the European Union, the use of both herbicides and fertilizers has remained fairly constant. Farmers are assumed to behave optimally, producing the best they can, given the agronomic constraints of their fields. Based on this assumption, reducing agrochemicals should inevitably have negative effects on food production, or reduce farmers’ incomes. Coupling empirical analysis based on field surveys and experimental trials where weed management and nitrogen input were manipulated in the same production fields and under real farming conditions, we demonstrate that high use of N fertiliser or intense weed control slightly increase yields, but that this increase is not enough to offset the additional costs incurred by their use. Our experimental design allowed inputs to be varied in a two-factor design, along a gradient spanning from organic to highly intensive farming, while holding all other conditions constant and thus avoiding confounding effects. Quantification of crop yields and gross margins from winter cereal farming showed that reducing dependence on weed management may not hamper cereal production in this system, and is economically profitable at the field level on the short term. Our study thus contributes to addressing a key gap in our economic knowledge, and gives hope for implementing win-win strategies for farmers and the environment.

## Introduction

Intensive agricultural systems have negative social and environmental externalities such as pollution^1^, affecting human health^2^, and biodiversity loss^3^, including the transformation of whole landscapes^4^. The regenerative capacity of entire ecosystems is now threatened, thereby endangering the very basis of the food supply for the world’s population^5^. Thus, a paradigm shift in modern agriculture is needed to make the world’s food production sustainable while mitigating these externalities. One, if not the major externality of intensive farming results from agrochemical inputs. In 2014, half million tons of pesticide (58% being herbicides) and 23 million tons of inorganic fertilizer (67% of which nitrogen) were used in Europe^6^. The environmental damage caused by excess nitrogen has been estimated to cost the European Union between €70 billion and €320 billion per year^1^. In 2003, the EU Common Agricultural Policy (CAP) incorporated a series of policy reforms to limit the environmental externalities of agrochemicals. Pillar 1 from the CAP primarily aims at stabilising farm income through direct payments to all farmers based on the amount of land owned and not on how much is produced^7,8^. While Pillar 2 is voluntary, refers to ‘extra’ payments based on social and environmental measures, and aims at achieving balanced rural development and sustaining an environmentally sound agriculture^7,9^. However, the quantities of both pesticides and inorganic fertilizers used over the last 15 years have at best remained constant^6,10^. France, being the fourth highest user of pesticides worldwide in 2014, adopted the ECOPHYTO plan in 2008 targeting a 50% decrease in pesticide use by 2018^11^. The plan failed^12^ and the deadline was postponed until 2025^13^.

The failure of current agro-environmental plans indicates that new public policies are required for reducing agrochemical consumption^2,14^, taking account of the complex, interdependent and conflicting forces between the environmental and socio-economic challenges^15,16^. Conjectures about profitability and risk-aversion are still the prime elements in farmers’ decision-making processes^17,18^. Farmers fear financial losses if they reduce agrochemical inputs^15,19^, although this has been questioned by recent evidence showing that there is no correlation between agrochemical use and productivity or profitability^20,21^. The assumption that farmers’ strategies are optimal for given environmental and agronomic constraints is currently untested, and there is no experimental evidence for this claim from rigorously designed experiments on farms^22^. Here, we specifically address two questions related to the use of (nitrogen) fertilizer and weed control, by farmers: i) do farmers maximize production, profitability or both? And, ii) can nitrogen and weed control be reduced with little or no costs to farmers?

**Figure 1:**
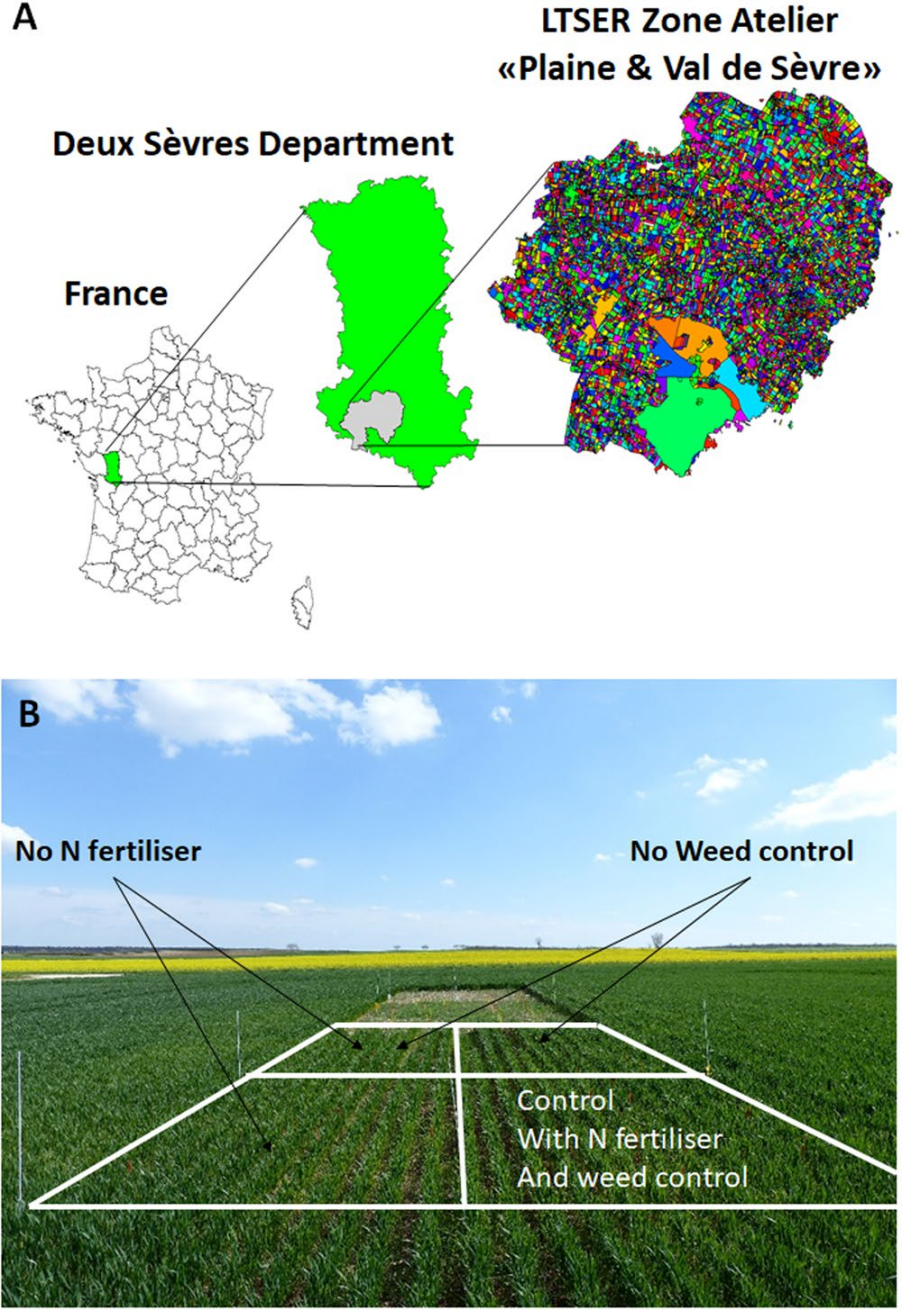
Study site, showing (**A**) the LTSER Zone Atelier “Plaine & Val de Sèvre”, and (**B**) the experimental design for the cropping season 2013/2014. The experimental area consisted in four zones, delineated by the two treatments and their interaction: no weed control, no nitrogen input, neither weed control nor nitrogen input, and control zone. In each experimental field, we had in place an area without crop for other consideration. More details on experimental design for the two cropping seasons are given in Supplementary Fig. 1.

Cereals and in particular wheat with a total production of ~730 Mt in 2014^23^, is one of the most important food staples. We focus on the use of two main farming practices, nitrogen (N) use and weed control through herbicides and mechanical weeding, for cereal production. These two practices are applied intensively because of the alleged benefits they provide, improving yields and reducing variability^19^. N inputs improve winter cereal yield steeply at low application rates but rather smoothly to flat at high application rates^24,25^, reduce competition by weeds^26^ and increase the protein levels in grain - as required by the food processing industry^27^. In this study, we intended to question the feasibility of lowering agrochemical use, in particular nitrogen and herbicides, without substantial changes in yield. With the help of farmers, we manipulated weed management and nitrogen inputs directly in their fields in a two-factor experimental design to investigate the effects of increasing or decreasing these two factors while holding all other conditions constant, such as soil class or other agricultural practices. The outcomes were assessed in terms of yield and gross margin in 55 fields in the LTSER Zone Atelier “*Plaine & Val de Sèvre*” in 2013 and 2014 (Fig. 1A; see also Supplementary Fig. 1). The fields belonged to 23 farmers, covering a wide range of soil properties and farming practices, from organic farming (OF) systems without pesticides and inorganic fertilizers, to intensive conventional farming (CF) systems with a high reliance on agrochemicals.

## Results

### Impact of nitrogen and weed control on winter cereal yield and economic returns based on survey data

**Figure 2:**
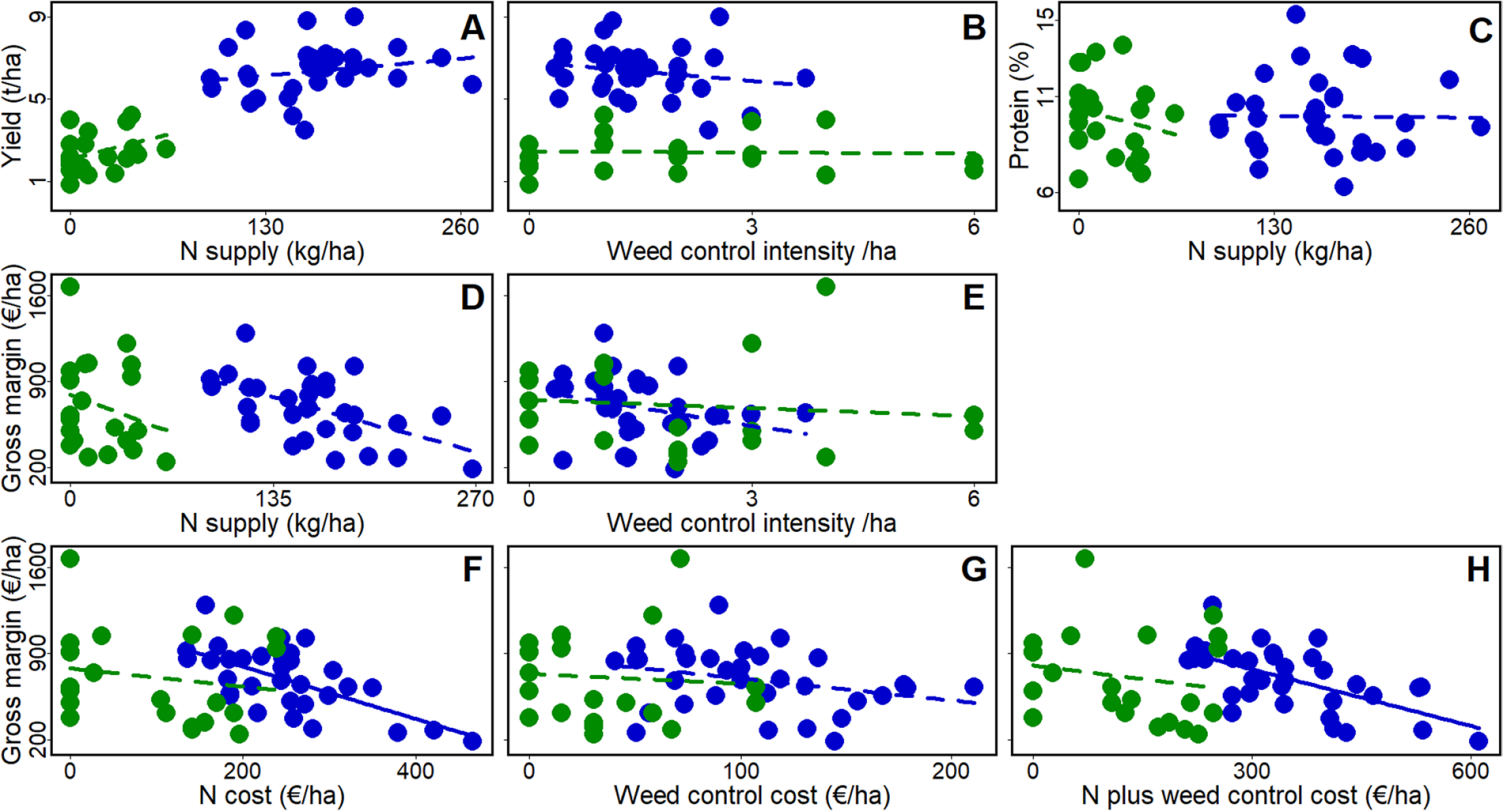
Effect of weed control and nitrogen management on crop yield (t ha^-1^), crop protein content (%) and gross margin (€/ha). Data were obtained from the farm surveys. Solid and dashed lines represent significant (P < 0.05) and non-significant relationships, respectively. The blue line is the regression for conventional farmers (CF), and the green line for organic farmers (OF). The cost of (organic plus inorganic) nitrogen and weed control includes in addition to product cost the application costs, i.e. machinery used in fertilizer application (for CF and OF), herbicide (for CF) and mechanical weeding (for OF). (**A**) Yield as a function of nitrogen dose, (**B**) weed control intensity (TFI for CF and number times the field was mechanically weeded for OF) and (**C**) Grain protein content as a function of nitrogen dose. Gross margins as a function of (**D**) nitrogen dose, (**E**) weed control intensity, (**F**) nitrogen cost, (**G**) weed control cost and (**H**) nitrogen plus weed control cost. Gross margins are computed without including Agro environmental measures subsidies (CAP pillar 2, see methods).

Field crop yields, farming practices and general information about the farm were collected by means of individual farmer interview after harvest (see Methods). Crop yield in conventional farming (CF) systems (6.3 ± 1.2 t ha^−1^, N = 33) was 2.6 times higher than in organic farming (OF) systems (2.4 ± 1.0 t ha^−1^, N = 22). This yield gap between CF and OF exceeds the values reported in the literature^28^ and is explained by differences in yield between crop species in the two farming systems (6.4 ± 1.2 t ha^−1^ in winter wheat which was mostly cultivated by CF farmers, versus 2.1 ± 1.3 t ha^−1^ and 2.3 ± 0.4 t ha^−1^ in spelt and triticale which were mostly cultivated by OF farmers). Further analyses therefore separated the two farming systems, but combined years since no year effect was detectable on yields (in CF: Student t-test, t = −0.28, *P* = 0.78; in OF: t = −0.66, *P* = 0.52). Our results did not reveal any significant increase of yield with N inputs, neither in CF (F = 1.475, d.f. = 31, *P* = 0.23) nor in OF (F = 3.302, d.f. = 20, *P* = 0.08; Fig. 2A). This may reflect input levels were close (or higher) to the levels maximizing yields. Yield was as well not affected by herbicide use in CF (F = 0.76, d.f. = 31, *P* = 0.39; Fig. 2B) nor by the number of times the fields were weeded mechanically in OF (F = 0.04, d.f. = 20, *P* = 0.85; Fig. 2B). Accounting for soil class, N residues in the soil, or weed control intensity did not change the relationships between yield and N or weed control (Supplementary Table 1). Similarly, grain protein content at the field level was unaffected by N input (for CF: F = 0.01, d.f. = 30, *P* = 0.94; for OF: F = 0.83, d.f. = 20, *P* = 0.37; Fig. 2C).

Crop yield was translated to gross margins, using the total revenue per field minus the respective variable costs (see Methods). The total revenue is composed by grain quantity, crop market price (different between OF and CF, and cereal variety) and quality (based on % grain protein). Despite lower crop market price for CF, due to higher yields, CF total revenue was c. 26% higher that OF (Supplementary Table 2). However, OF showed variable costs approximately half that of CF (Supplementary Table 2), which resulted in a 3% higher gross margins for OF. Gross margins decreased with increased N supply (Fig. 2D) and weeding (Fig. 2E), and we found as well a negative correlation between gross margins in CF fields and N costs (F = 21.92, d.f. = 31, *P* < *0.01*; Fig. 2F) and weed control cost (F = 3.08, d.f. = 31, *P* = 0.09; Fig. 2G). These results indicate that CF was less profitable in more intensively farmed fields. Conversely, no significant negative correlation between gross margins and N costs was found in OF (Fig. 2H). The robustness of these empirical results was checked against CAP subsidies for agro-environmental measures (i.e. pillar 2, specifically reduction of herbicides and/or N, or organic farming) and various scenarios for wheat market and organic fertilizers prices (see Supplementary Table 3 for details). All yielded very similar results, although CAP subsidies (both pillars 1 and 2) accounted for 51% and 57% of CF and OF farmers’ gross margins, respectively (Supplementary Fig. 2). In absence of CAP pillar 1 subsidies, 15% and 10% of CF and OF farmers, respectively, had negative gross margins (Supplementary Fig. 2).

### The effect of experimental input reductions on winter cereal production and economic returns

It is possible that, despite the negative relation between the gross margins and the intensiveness in CF, farmers still do their best, and the lower gross margin is simply a result of confounding factors such as lower quality soils or environmental conditions. To avoid such possible confounding factors, and determine whether farmers maximize production, gross margin, or both, we experimentally manipulated N input and weed control in the same 55 fields with the help of the farmers. Here N input and weed control intensity were handled in a two-factor experimental design with two or four combinations of N and weed control treatments (see Methods), allowing treatment comparisons within fields in a paired design comparison. Hence we quantify the effect of the treatment on yield (gross margin) to the yield (gross margin) obtained in the control situation within the same field by computing dimensionless effect size ratio.

**Figure 3:**
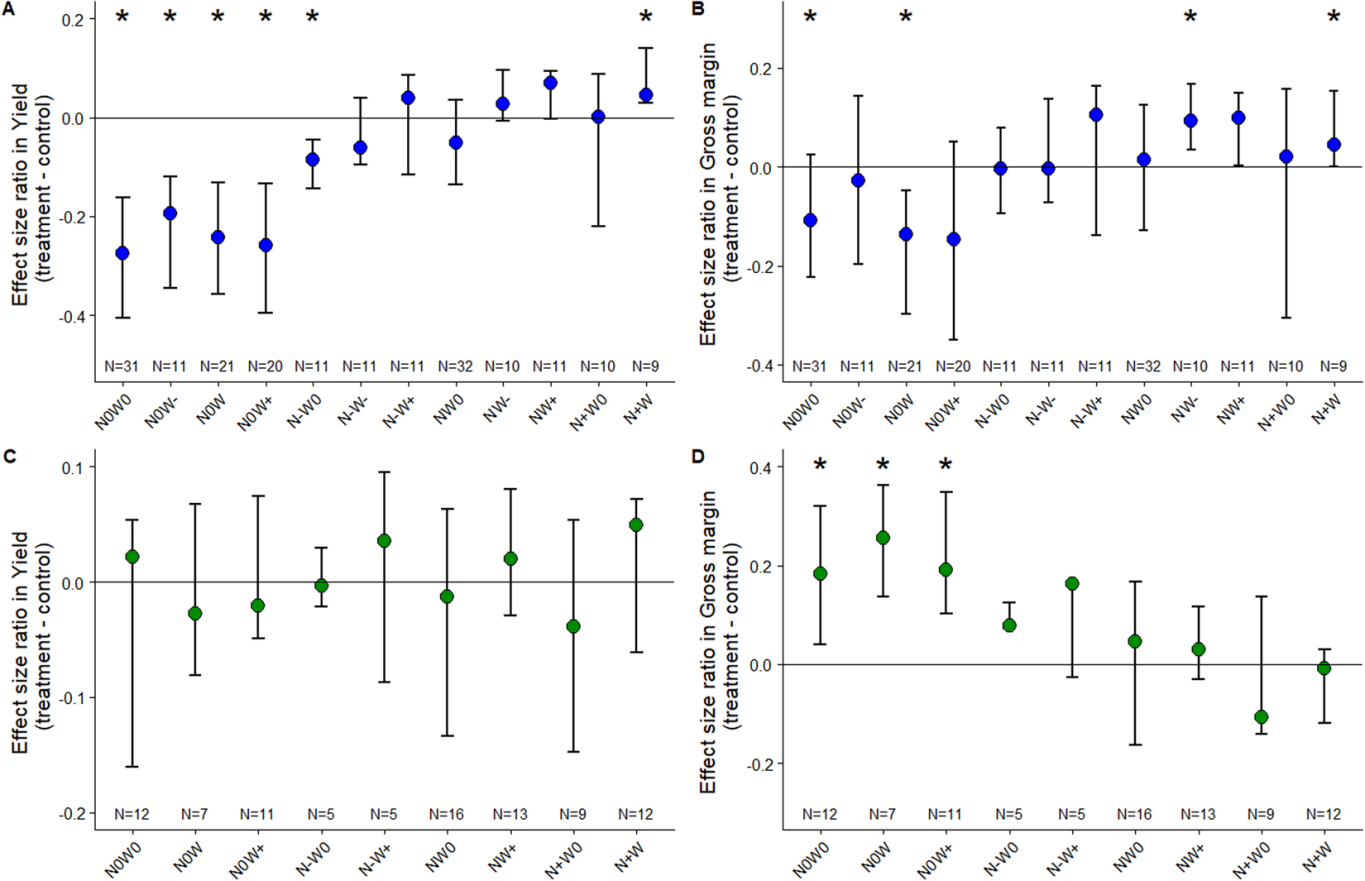
Effect size ratio in yield (left column) and gross margin (right column) for each experimental treatment for conventional (CF; blue) in (**A,B**), and organic (OF; green) farming in (**C,D**) (medians and 25% and 75% quantiles bars). Effect size ratio quantifies the proportional difference between crop yield or gross margin in control and treatment. A negative value indicates that yield or gross margin is higher in control than in treatment, while a positive value indicates that yield or gross margin is higher in treatment than in control. Stars indicate the treatments that were significantly different from the control (Sign tests). N0, N−, N and N+ indicate no nitrogen, reduced, current (i.e. farmers’ current practice) and increased nitrogen application treatment, respectively. W0, W− and W indicate no, reduced, and current (i.e. farmers’ current practice) weed control respectively. W+ is a treatment with the farmers’ current weeding control supplemented by an additional hand weeding treatment. Number of observations per treatments is shown at the base of each column.

In CF, significant differences in yields (Sign test statistics, see Methods) were only observed when N inputs were stopped whatever the weed control treatments, or when N inputs were halved with no weed control treatment (Fig. 3A). Yields significantly decreased on average by 24.5% (±1.8) when only N inputs were stopped, and by 14.0% (±10.5) when only herbicide use was stopped. In all other situations, no significant differences in yields were observed (Fig. 3A), supporting the empirical findings that no significant relationships between N input and yields were observed among CF.

The control treatment (farmer’s normal dose of N and herbicide) produced on average equal or higher yield than any level of N or herbicide reductions, alone or in combination. However, significantly higher gross margins were detected for reduced weed control when increasing or maintaining N input, or when increasing N input while keeping weed control treatment (Fig. 3B). For OF, no significant differences in mean yields were found between control and any experimental treatment (Fig. 3C). Because of less production costs when N input or weed control were stopped, higher gross margins were therefore found in OF with a significant increase of in average 21.4% (Fig. 3D).

Whether farmers maximize their production rather than gross margin is still debated^29^. We therefore examined individual farmers’ strategies by comparing their observed field management practices (using control plots) with each N input and weed control treatment (using experimental plots) per field and per farmer. Given that strong reduction in N supply (in our experimental design, either reduced or stopped) resulted in significant decrease of yield and/or gross margin in CF, we focused here on weed control reduction alone or in combination with N supply reduction.

**Figure 4:**
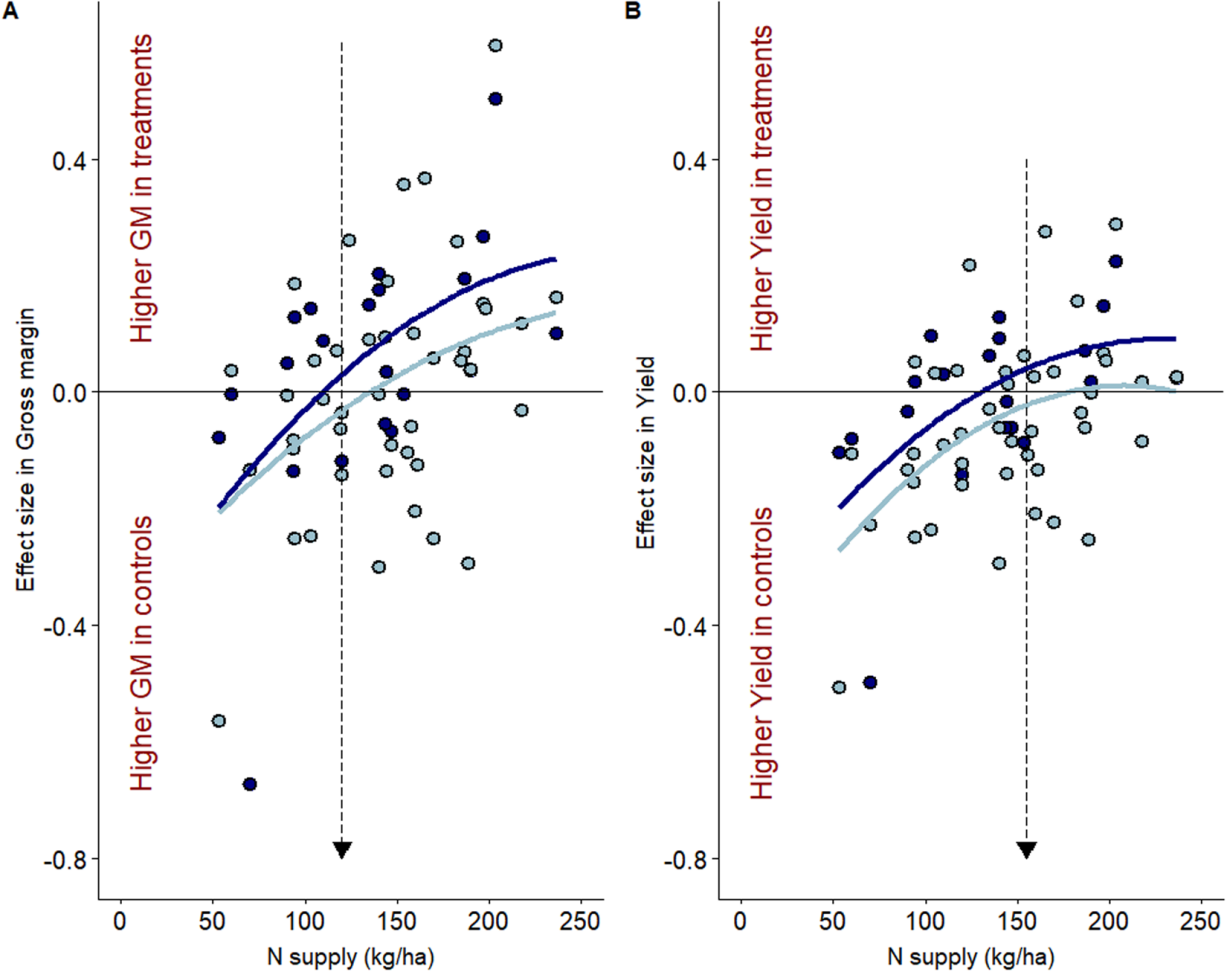
Relationship between the effect size ratio in Gross margin (**A**) and Yield (**B**), and farmers’ N supply in CF fields. Effect size ratio were computed between control and (i) weed control reduction with no change in N supply (NW0/NW−) or (ii) weed control reduction when N supply is reduced (N−W0/N−W−). Treatments are indicated by the different colours: light blue for N−W0/N−W−, and dark blue for NW0/NW−. Lines show saturated relationships. Arrows highlight N supply threshold over which reducing weed control alone or with N supply benefits gross margin (**A**) and yield (**B**). Positive values indicate that GM (**A**) or Yield (**B**) are higher in treatment plots than in control ones. While negative values indicate that higher gross margins or yields are obtained under farmers’ current strategies (controls). Relationships between the effect size ratio in Gross margin and Yield, and farmers’ N supply in OF fields are shown in Supplementary Fig. S4).

In CF, in fields with low N supply (below 120 kg.ha^−1^), a further reduction of either N supply or weed control resulted in a decrease in yield, gross margin, or both (Fig. 4A,B). However, when farmers were using high N supply (higher than c. 120–140 kg.ha^−1^ Fig. 4A), we found that reducing weed control resulted on average in higher gross margin, whether N supply was reduced (light blue) or not (dark blue, Fig. 4A). The increase of gross margin induced by the reduction of weed control was higher in the fields with the higher N supply. Reducing weed control did not alter yield when N supply was kept constant in fields with high N supply (higher than 120 kg.ha^−1^; dark blue, Fig. 4B). Reducing both weed control and N supply did not even reduce yield in fields with N supply higher than 160 kg.ha^−1^ (light blue, Fig. 4B). In OF, reducing weed control or both N supply and weed control did not significantly affect farmers’ gross margin while maintaining crop yield (Supplementary Fig. S4). We finally used experimental treatments to find the optimal combination of N supply and weed control treatments that would maximize either margin or yield. Although results should be taken with caution since they are based on 1 m² quadrats, we found that among 23 farmers, in at least one of their fields, nine maximized simultaneously their production and gross margins, seven maximized solely their production, five their gross margins and two did not maximize either production or gross margin (Supplementary Table 4). Overall, with the best economic strategies, the average gross margins were almost identical between OF and CF (Supplementary Table 5).

## Discussion

Our experiment in real farming conditions provides evidence that, in most of the intensively managed fields, reducing weed control and to a lesser extent, N inputs, can increase farmers’ gross margins with no change in yield. Although obtained from fairly small experimental plots, these results are in full agreement with previous studies which empirically showed that reducing N inputs or herbicides (25–30% on average) had no effect on production or profitability^14,21,30,31^. High level of N residual in soil in both conventional (69.2 ± 39.6 kg.ha^−1^) and organic (36.6 ± 31.2 kg.ha^−1^) fields at the beginning of the cropping season, as well as the low differences in weed density (44.9 ± 29.22 plants per m²)^32^ between reduced weed control treatments and controls may explain why these reductions are not detrimental. Moreover, the density of weeds (112.19 ± 139.97 plants per m² on average in no weed control plots^32^) was rather low and consistent with the major decline of weed species in the seed bank documented for intensively managed agroecosystems in recent decades in most West-European countries^33,34^. Finally, crop plants alone were shown to strongly reduce weed biomass (almost 65% in average^32^). All together, these elements alleviate potential substantial increase of weeds in subsequent years after decreasing herbicide use.

A prerequisite to efficiently address key issues, such those derived from agro-chemical contamination, commonly shared by farmers, policy makers and population in general, is to develop sound scientific knowledge to help identify future policy alternatives. To our knowledge, our study is the first to explicitly test the effects of reductions in agrochemicals on production and profitability, in real conditions, in the same soil with the same farming management strategies, exploring a wide range of soil class and management intensities. A classical argument relates to potential yield loss if we were to adopt measures reducing drastically the use of agrochemicals. However we empirically demonstrate that such losses are not necessarily observed. Although our results are based on modest sample size and show large confidence intervals on some of the estimated effect sizes (thus we cannot exclude that the lack of significant difference results from a lack of power), our results are in line with a large body literature warning to the overuse of nitrogen^19,35,36^ and herbicides^20,26,37,38^. According to our experimental results, in our region, conventional farmers are relying highly on agro-chemicals (c. 160 N kg.ha^−1^ and 2.7 Treatment Frequency Index (TFI) in average^39^), while N supply and TFI herbicide are strongly correlated (Supplementary Fig. 3). Our results suggest that 120–130 N kg.ha^−1^ (a reduction on average of 20%, thus slightly below of the 50 kg.ha^−1^ reduction suggested by Van Grinsven *et al*.^40^) and a 50% decrease (in line with Gaba *et al*.^20^ and Lechenet *et al*.^21^) in weed control TFI (Supplementary Table 5) would maintain yield and increase gross margin, in line with Ecophyto Plan 2 targets^13^. These are, however, average values: our results indicate that farmers already using low nitrogen supply would reduce their gross margins if reducing N supply, but farmers currently using high N supply (and high herbicide treatments) would improve their margins by substantially reducing herbicides and to some extent, N supply. In organic farms, mechanical weed control may be reduced by half (Supplementary Table 5), which might as well improve soil conservation^41^ and decrease CO_2_ emissions due to less runs with machinery. Although not been quantitatively assessed until now, but urgently needed, we believe it is possible that similar results could be obtained in other crops.

Reducing N input and weed control could therefore be possible without increasing the complexity of farming management and decision-making, which are the main hurdles in the way of change^42^. Our research has strong implications for policy makers and practitioners, particularly regarding the goals to reduce pollutant emissions, including nitrogen oxides, proposed by the ratified Gothenburg Protocol^43,44^ and the repeated debates and arguments concerning pesticide use and possible bans^37,45,46^ (e.g., neonicotinoids, glyphosate). Our results corroborate previous studies^20,21,47^ that failed to detect a positive effect of herbicides on yield, and shows the first practical evidence that a reduction is feasible and would bring actually cost savings to farmers. Our study underlines (i) the possibility to partly reduce N input and weed control without significant yield loss but with higher economic return, (ii) the importance of estimating the agricultural and economic consequences of agrochemical reductions and (iii) the need to collaborate with decision makers in real farming conditions to facilitate result dissemination. However, we acknowledge that these effects need to persist at larger spatio-temporal scales to guarantee farmers’ acceptance and long-term productivity. Future research is now urgently needed to evaluate the ecological and economic feasibility of reducing agrochemicals, in various geographic areas, at larger temporal (a full rotation period) and spatial scales (field or farm level), including other crops that are intensively managed, such as oilseed rape, maize and sunflower.

## Methods

### Data reporting

No statistical methods were used to predetermine sample size. Farmers were not randomly chosen, but rather the sample was selected based on the farmer’s willingness to do the experiments. We did however stratify our sampling design in relation to farming system (see below), farm size (ranging from 18 to 480 ha, Supplementary Table 2), and soil class (varying from very poor dry soil 20 cm deep, to 50 cm silt) classified here as superficial (12 CF and 8 OF), medium (15 CF and 4 OF) and deep soils (6 CF and 10 OF). Measurements of yields were carried out blind.

### Study area

The study took place in the LTSER “*Zone Atelier Plaine & Val de Sèvre*” (http://www.za.plainevalsevre.cnrs.fr/)^48^, a long term social-ecological research site of 450 km² located in central western France, in the south of Deux-Sèvres district in the Nouvelle Aquitaine region (46.23°N, 0.41 W). It is an agricultural landscape dominated by intensive cereal crop production (44% winter cereals, most of which are winter cereal cultivars, 8–12% rape seed, sunflower or maize, and 14% meadows and alfalfa, 4% woods and 9% villages; all data from 2010 season), with an average field size of 4–5 ha.

### Farming system description and sampling design

For this study, the periods of interest were the winter farming seasons October 2012–July 2013 (16 fields) and October 2013–July 2014 (40 fields). Only one OF field, in 2012/1013, was entirely excluded from the analysis due to the total yield loss caused by a hailstorm. The sample comprises a total of 23 farmers (55 fields) that participated and carried out the experiment in real field conditions. In the first cropping season, 14 farmers participated in the experiment. In the second cropping season, 11 farmers remained in the experiment with the participation of eight new farmers. Two main groups of agricultural farming system were considered: (i) conventional farming (CF) with 14 farms (33 fields) using conventional chemical crop fertilization and pest control techniques; ii) organic farming (OF) with nine farms (22 fields) complying with a set of farming rules specified in EU Regulation (EC) No 834/2007 and its implementing regulations (EC) No 889/2008, such as the restriction of inorganic fertilizers and chemical pesticides. Four winter cereals were used by farmers: winter wheat (32 CF and ten OF fields), barley (one CF and four OF fields), spelt (four OF fields) and triticale (four OF fields). Here, we combine the four different species and use the broad term winter cereal, since their agronomic characteristics are very similar.

### Field experimental design

The experiment consisted in manipulating the nitrogen (N) inputs and weed control intensity (herbicide in conventional and mechanical weeding in organic farms), comprising a total of 510 experimental subplots (including controls). The control treatment was the normal practices of the farmer in terms of N input (quantity and type) and weed control. We set up a split-plot design with two factors allowing the effect of each factor alone and their interactions to be assessed. The experimental design varied slightly between the two seasons. In the 2012/2013 season, the experiment consisted of five experimental plots of 200 m², two in the field margins (here, the first five meters in the field) and three in the centres (Fig. 1B). Each plot was divided in two experimental subplots in which two treatment combinations were put in place: control and no N input (see Supplementary Fig. 1). Weed control was manipulated in only one of the two plots at the field margin. Therefore, in the three centre plots and in one of the field margin plots, the weed control was the farmers’ usual practice and only two experimental combinations were present (control and no N input). In the field margin plots with no weed control, the experimental combinations were N input and no weed control and neither weed control nor N input.

In 2013/2014 season, the experiment consisted of one 200 m² plot, equally divided in four subplots, in the centre of the 40 fields. Each subplot consisted in one of the four treatment combinations: control, no weed control, no N input, and neither weed control nor N input. In addition, we added two treatment levels for N inputs and for weed control application, corresponding either to a reduction (‘lower dose’) in half of the fields or an increase of dose (‘extra dose’) in the other half. These supplementary treatments were applied in quadrats within the four subplots (Supplementary Fig. 1). The reduced dose implied that one of the N applications or weed control treatments was eliminated. The extra dose for N was an extra 50 kg/ha of N (in the form of Urea) applied in conventional fields and 42.7 kg/ha of N (in the form of goat manure) in organic fields. The ‘extra dose’ for weed control application was the farmers’ normal weed control supplemented by additional hand weeding (see Supplementary Fig. 1 and Table 6 for a detailed description of the inputs for each treatment).

### Farmers’ survey

Information about crop yields and farming practices (pesticide and fertilizer use, ploughing and mechanical weed control system) and general information about the farm (number of crops, agricultural equipment) was collected by means of interviews with all the individual farmers after harvests. Crop yields from interviews were not available for three (three fields) and five (seven fields) farmers in 2013 and 2014 (see below the estimation procedure). The general statistics obtained from the questionnaires is presented for each farming system in Supplementary Table 2. From the farm surveys, we derived weed control indicators, nitrogen input values, and yield estimates.

#### Weed control intensity

Two indicators were used for weed control intensity according to the farming system. In CF, we used the Treatment Frequency Indicator (TFI) that quantifies the number of recommended doses applied to each unit of cropped area. TFI per hectare was expressed as:

1 $$TFI=\sum _{j=1}^{k}(\sum _{i=1}^{n}\frac{{D}_{i}.{S}_{i}}{D{h}_{i}.{S}_{t}})$$

where *D*_*i*_, *D*_*hi*_ and *S*_*i*_, i = 1, …, n are, respectively, the applied dose, the national recommended dose, and the treated surface area for the n spraying operations; and *S*_*t*_ is the total field area (see Kudsk & Jensen^49^). This includes all the pesticides treatments applied in a given crop field (except for seed treatment). The reference dose is provided for a given pesticide commercial product and corresponds to the minimum registered dose for a given crop. In OF, we used the number of mechanical weeding operations. Mechanical weed control operations were performed prior to the establishment of the crop as soil tillage, such as ploughing or disking, or after the establishment of the crop in the crop row with specific equipment, such as rotary hoes. Over our sample of farms, TFI ranged from 0 to 3.7 and mechanical weeding operations from 0 to 6.

#### Nitrogen input

The nitrogen used by farmers can be divided in two major groups: inorganic and organic fertilizers. Since inorganic nitrogen is rapidly available to plants, the quantity of nitrogen used was directly calculated according to the fertilizer composition and the respective quantity applied. Conversely, organic nitrogen is a relatively stable compound and it has to go through mineralization in order to be converted into its inorganic forms. Thus, quantity of nitrogen mineralized in the organic fertilizers was calculated (see^50^ for a detailed description).

#### Yield values

Crop yield and quality were assessed using grain yield biomass and grain protein content measured in a 1 m² quadrat. In both cropping seasons, grain yield was estimated by harvesting 1 m² quadrat per experimental treatment (16 1 m² quadrats in 2012/2013 and 40 1 m² quadrats in 2013/2014). In 2013/2014, an additional 1 m² quadrat per field (40 in total) was also harvested outside the experimental plot to control for experimental bias and its values used as control plots. Harvests were performed one week before the fields were harvested by the farmers. Samples were oven-dried at 80 °C for 48 h and weighed. Samples were then analysed for their C and N contents by dry combustion with an automatic C/N-Analyser (reference method ISO 10694 & 13878, Forest Research, UK) in order to estimate grain protein concentration (%N). Weed pressure as well as crop yield are known to vary between the margins and cores of fields^51^. Consequently, we performed a preliminary analysis to relate crop yields measured in experimental plots in field margins to the yields measured in the field cores. Two linear models (LM), one per farming system, were used to estimate the relationship between crop yields in the two field compartments for the normal weed control and nitrogen input and normal weed control with no nitrogen input experimental treatments. The estimated slopes of the two models were used to adjust field margin to the respective field core yields (for CF: *a* = 0.715; *R*^2^ = 0.82*, F* = 186.2*, d.f*. = 40*, P* < 0.01; for OF: *a* = 0.989; *R*^2^ = 0.64*, F* = 23.9*, d.f*. = 12*, P* < 0.01). Crop yields were also obtained using data from the surveys for the empirical analysis. Crop yields from the surveys of the farmers were missing for ten fields. For these fields, we estimated crop yields using the yield measurements in the control plots. For each field, we first calculated the average crop yield values obtained in control plots in the field core (three in 2013 i.e. in the three centre plots and in 2014, two i.e. one in the experimental plot and one outside, or the one outside for halved dose treatment fields). A linear model was calibrated using crop yield from farmers’ surveys as the response variable and average experimental crop yield values per farming system as the explanatory variables. Both models had a high explanatory power (for CF: *a* = 0.839, R^2^ = 0.94, *F* = 466.3, *d.f*. = 30, *P* < 0.01; for OF: *a* = 0.849, R^2^ = 0.95, *F* = 309.8, *d.f*. = 14, *P* < 0.01). The output from these models was used to estimate the missing values of crop yield considering their respective farming system. We tested the effect of including estimated values in our analysis and found similar results with and without including them (data not shown). Representativeness of crop yield harvested in the 1 m² plots was tested against field crop yield obtained from farmers’ survey using a linear mixed model with intercept zero and field ID as a random factor. We found a strong relationship between the two estimates of yield (R²_m_ = 0.69 and R²_c_ = 0.85, χ² = 180.43, d.f. = 1, *P* < 0.0001).

### Evaluation of gross margins

Farmers’ relative profitability was assessed using gross margins (GM) to allow for comparison of economic efficiency across farmers’ management strategies assuming that fix costs, i.e. land, machinery and equipment requirements, are similar. The GM per hectare is calculated accounting for the final grain yield revenue plus CAP Pillar 1 subsidy minus variable costs (VC; details below) associated with the agricultural activity (winter cereal production). Note that the GMs do not include the CAP pillar 2 (Agri-environmental Measures, AEM) subsides. However, since this may also affect yield and farmers may depend on this extra revenue to be economically viable, we have also evaluated their weight on GMs in a robustness test (Supplementary Table 3 and Fig. 2). GM was calculated as follows:

2 $${\rm{GM}}=[{\rm{g}}({\rm{GP}})\,\cdot \,{\rm{GY}}]+{\rm{CAP}}-{\rm{VC}}$$

where GP is the grain protein content (%), g is the function of GP for the price of wheat (€ t^−1^), GY is the grain yield (t ha^−1^) and VC are the variable costs (€ ha^−1^).

#### Grain prices

Grain prices for 2013 and 2014 were set equal to the average annual market values for CF and the average cooperative price for OF (values and references for the prices are given in Supplementary Table 7). Prices are commonly increased by 3€ t^−1^ or decreased by 4€ t^−1^ per 1% of protein content above or below a level of 11%^52^.

#### Fertilizer cost

Fertilizers costs per hectare were calculated according to their total composition in nitrogen (0.9 € ha^−1^), phosphorus (0.95 € ha^−1^), potassium (0.65 € ha^−1^) and sulphur (0.76 € ha^−1^), the quantity applied and cost of each respective application. Cost of organic fertilizers were different from those of mineral fertilizers. For organic fertilizer the total composition refers to quantity of organic nutrients prior to mineralization. For inorganic N, calculations were directly done on its inorganic form value. Organic fertilizers may come from and be used in the same farm. In that case, the same prices described above were considered due as opportunity costs, i.e. the farmer could have sold its fertilizer and purchase an equivalent elsewhere.

#### Pesticide cost

Pesticide costs per hectare were calculated according to their market price, obtained from cooperative prices, the quantity applied and cost of each respective application.

#### Variable costs (VC)

VC include all input costs that vary with one crop production cycle, such as seeds variety, soil tillage, fertilizers and pesticides (including application costs), and mechanical weeding (references for the prices of inputs and machinery are given in Supplementary Table 8). For the same quantity of output, VC can fluctuate depending upon the amount or type of inputs used (e.g. the large range of weed management options).

#### Nitrogen and weed control costs

N costs were obtained directly from the fertilizer costs (including application costs). Weed control costs for CF were obtained by isolating the herbicide cost (including application costs) within the total pesticide cost. For OF, the total cost (including frequency) of mechanically removing weeds was used.

### Statistical analyses

The effect of N input and weed control (using TFI for CF and the number of time the fields were mechanically weeded for OF) on yields from the survey of farmers were analysed using a linear model with N input (or weed control) and farming system as factors as well as their second-order interactions. We further tested if accounting for soil class, N residues in the soil, or the interaction between N inputs and weed control intensity improved the model, by adding each variable in separate linear models as a single effect and in interaction with the farming system (see Supplementary Table 1a). This strategy was selected to ensure adequate statistical power since the sample sizes did not allow models with many factors. The same procedure was applied for testing the effect of N and weed control costs on gross margins. We then explored whether reducing N supply, weed control or both, holding all other conditions constant could benefit to farmers (resulting in higher gross margins) without a major crop yield loss. We therefore performed paired comparison of yield and GM within each field. We first computed dimensionless effect sizes for the impact of weed control and N supply manipulation on crop yield (GM) measured as ratio, which quantifies the proportional difference between crop yield (GM) in control and treatment. The ratios were computed as the difference between the crop yield (GM) in treatment and control over the sum of the crop yield (GM) in control and treatment, hence they varied between −1 and 1. A negative value indicates that, in a same field, crop yield (GM) is generally higher in the control plot than in the treatment one, while a positive value shows higher crop yield (GM) in the treatment plot than in the control one. We used sign tests to test for significant differences per treatment. Finally, we explored the situations in which weed control could be reduced, while maintaining or reducing N supply. We focused on the relationship between N supply and the effect size computed between control and treatments in which weed control was reduced or stopped. Weed control reduction treatments were combined by N supply modalities. Relationships were explored using quadratic regression models for CF as we expected a saturated relationship and simple regression models for OF where the range of N supply values was lower. All statistical analyses were performed using R 3.1.1^53^.

## Supplementary information

SUPPLEMENTARY INFO

## Data Availability

The datasets analysed during the study are available from the corresponding author upon request.

## Code Availability

The computer code used for this study is available from the corresponding author upon request.
