# Supplementary material for: Experimental and empirical evidence shows that reducing weed control in winter cereal fields is a viable strategy for farmers
Source: Sci Rep. 2019 Jun 21;9:9004. doi: 10.1038/s41598-019-45315-8 (PMC6588622; doi:10.1038/s41598-019-45315-8)
Supplement: Supplementary file 1 — SUPPLEMENTARY INFO [file 41598_2019_45315_MOESM1_ESM.doc]

# Supplementary information related to manuscript: Contains Supplementary Tables, Supplementary Figures and Additional references in Supplementary Information.

**Experimental and empirical evidence shows that reducing weed control in winter cereal fields is a viable strategy for farmers**

**Rui Catarino^1^, Sabrina Gaba^2,3^ & Vincent Bretagnolle*^1,3*^**

^1^ CEBC, UMR 7372, CNRS & Université de la Rochelle, Villiers-en-Bois F-79360, France

^2^ USC 1339 Centre d’Etudes Biologiques de Chizé, INRA, F-76390 Villiers-en-Bois, France

^3^ LTSER “Zone Atelier Plaine & Val de Sèvre”, CNRS, Villiers-en-Bois F-79360, France

^*^ breta@cebc.cnrs.fr

# Supplementary Tables

## Supplementary Table 1: Yield in response to N input and Weed control intensity. One additional factor (Soil, Year and either Weed management or N input) is added at a time to test whether the model is improved and/or the effect of N input (or weed management) is affected by the additional factor. β is the coefficient for nitrogen input or weed control intensity.

|  | Conventional farming | | | |  |  | Organic farming | | | |  |
| --- | --- | --- | --- | --- | --- | --- | --- | --- | --- | --- | --- |
| Models | DF | F | AIC | β | *P* |  | DF | F | AIC | β | *P* |
| Nitrogen input |  |  |  |  |  |  |  |  |  |  |  |
| 1. N_input_ | 1 | 113.1 | 109.7 | 0.01 | 0.23 |  | 1 | 3.3 | 63.1 | 0.02 | 0.08 |
| 1. N_input_ + S_class_ | 3 | 114.2 | 113.6 | 0.01 | 0.24 |  | 3 | 1.8 | 64.8 | 0.02 | 0.10 |
| 1. N_input_ + Year | 2 | 118.8 | 111.7 | 0.01 | 0.25 |  | 2 | 1.6 | 65.0 | 0.02 | 0.10 |
| 1. N_input_ + N_Soil_ | 2 | 120.5 | 110.6 | 0.01 | 0.16 |  | 2 | 1.6 | 65.0 | 0.02 | 0.09 |
| 1. N_input_ + WCI | 2 | 112.8 | 108.7 | 0.01 | 0.09 |  | 2 | 1.6 | 65.0 | 0.02 | 0.09 |
|  |  |  |  |  |  |  |  |  |  |  |  |
| Weed control intensity | |  |  |  |  |  |  |  |  |  |  |
| 1. WCI | 1 | 112.6 | 109.9 | -0.30 | 0.27 |  | 1 | 0.0 | 66.4 | -0.02 | 0.88 |
| 1. WCI + S_class_ | 3 | 115.1 | 113.4 | -0.36 | 0.22 |  | 3 | 0.9 | 67.4 | -0.10 | 0.44 |
| 1. WCI + Year | 2 | 117.9 | 111.2 | -0.41 | 0.18 |  | 2 | 0.1 | 68.2 | -0.03 | 0.80 |
| 1. WCI + N_Soil_ | 2 | 120.6 | 111.3 | -0.32 | 0.25 |  | 2 | 0.1 | 68.3 | -0.02 | 0.85 |
| 1. WCI + N_input_ | 2 | 120.0 | 108.7 | -0.47 | 0.10 |  | 2 | 1.6 | 65.0 | -0.03 | 0.77 |

Tables shows the output of the models for yield response to the quantity (kg ha^-1^) of nitrogen applied (N_input_) and weed control intensity (WCI) accounting for a parameter known to affect yield in the model. The analysis aimed to examine whether accounting for a parameter known to affect yield in the model would improve the model and modify the relationships between nitrogen applied or weed control intensity and yield. Because of the small sample size, variables were added one-by-one. We investigated the effect of four variables: year (2 levels: 2013 and 2014), soil class (S_class_, 3 levels: superficial, medium and deep soils), nitrogen residues in the soil (N_Soil_) and either nitrogen applied or weed control intensity depending on the relationship studied. We observed that accounting for other variables does not improve the model for yield. Results for conventional (CF) and organic (OF) farming are shown.

## Supplementary Table 2: General statistics obtained from the questionnaires for both conventional and organic farming.

|  | CF | | | OF | | |
| --- | --- | --- | --- | --- | --- | --- |
|  | mean (±SD) | min | max | mean (±SD) | min | max |
| Farm area (ha) | 203.6 (±102.2) | 63.0 | 480.0 | 111 (±41.2) | 18.0 | 148.0 |
| Wheat area (ha) | 62.2 (±28.5) | 10.0 | 140.0 | 13.4 (±7.9) | 2.7 | 30.0 |
| Yield (t ha^-1^) | 6.3 (±1.2) | 3.5 | 9.0 | 2.4 (±1) | 0.8 | 4.2 |
| Total Revenue (€ ha^-1^) | 1441.2 (±241.7) | 901.8 | 1855.8 | 1142.3 (±372.8) | 590.0 | 1912.0 |
| Gross Margin (€ ha^-1^) | 690.4 (±252.5) | 190.4 | 1293.8 | 711.3 (±366.9) | 246.8 | 1667.1 |
| N costs (€ ha^-1^) | 249.5 (±76.6) | 135.2 | 466.3 | 94.5 (±88.8) | 0.0 | 238.8 |
| Weed control costs (€ ha^-1^) | 105.3 (±43.2) | 40.6 | 211.0 | 32.5 (±33.3) | 0.0 | 107.4 |
| Total Variable costs (€ ha^-1^) | 750.8 (±193.3) | 434.2 | 1097.1 | 430.9 (±205.8) | 130.1 | 763.7 |

General statistics obtained from the questionnaires for both conventional (CF, N=33) and organic farming (OF, N=22). Farm area, wheat area and yield values were obtained directly from the farm questionnaires. The remaining values were calculated as described in the Methods.

## Supplementary Table 3: **Robustness test under seven different** economic **scenarios.**

|  |  | Grain prices  (€ t^-1^) | | | | | | | | | | | | | | | |  | Organic fertiliser prices  (€ kg^-1^) | | | | |  | CAP  (€ ha^-1^) | |
| --- | --- | --- | --- | --- | --- | --- | --- | --- | --- | --- | --- | --- | --- | --- | --- | --- | --- | --- | --- | --- | --- | --- | --- | --- | --- | --- |
|  |  | CF | | | | | | | | OF | | | | | | | |  |  | | | | |  | CF | OF |
|  | | Winter wheat | | Barley | | Spelt | | Triticale | | Winter wheat | | Barley | | Spelt | | Triticale | |  | N | P | K | S | Mg |  |  | |
|  | | 2013 | 2014 | 2013 | 2014 | 2013 | 2014 | 2013 | 2014 | 2013 | 2014 | 2013 | 2014 | 2013 | 2014 | 2013 | 2014 |  | 0.90 | 0.95 | 0.65 | 0.76 | 0.20 |  | 300 | 300 |
| Baseline scenario | | 215 | 187 | 195 | 160 | - | - | - | - | 363 | 367 | 395 | 395 | 390 | 390 | 289 | 289 |  |  |  |  |  |  |  |  |  |
| Scenario 1 | |  |  |  |  |  |  |  |  |  |  |  |  |  |  |  |  |  |  |  |  |  |  |  | 300 to 521 | 537 |
| Scenario 2 | |  |  |  |  |  |  |  |  |  |  |  |  |  |  |  |  |  |  |  |  |  |  |  | 0 | 0 |
| Scenario 3 | | 201 | 201 | 177 | 177 | - | - | - | - | 365 | 365 | 395 | 395 | 390 | 390 | 289 | 289 |  |  |  |  |  |  |  |  |  |
| Scenario 4 | | 187 | 167 |  |  |  |  |  |  |  |  |  |  |  |  |  |  |  |  |  |  |  |  |  |  |  |
| Scenario 5 | | 250 | 196 |  |  |  |  |  |  |  |  |  |  |  |  |  |  |  |  |  |  |  |  |  |  |  |
| Scenario 6 | | 209 | 193 |  |  |  |  |  |  |  |  |  |  |  |  |  |  |  |  |  |  |  |  |  |  |  |
| Scenario 7 | |  |  |  |  |  |  |  |  |  |  |  |  |  |  |  |  |  | 0 | 0 | 0 | 0 | 0 |  |  |  |

Three factors play a major role in the difference between CF and OF gross margins (GM): CAP subsidies, grain prices and fertilizer prices. We explored the robustness of GM to prices using seven scenarios in which these three factors were modified. The table shows the parameters used in the robustness test for the seven different scenarios for conventional (CF) and organic farming (OF). The seven scenarios were: Scenario 1: inclusion of CAP pillar 2 (agro-environmental measures subsidies), which depends on the measure undertaken by farmers: herbicide reduction (84€ ha^–1^), N fertilizer reduction (137€ ha^–1^) and organic farming (227€ ha^–1^); Scenario 2: no CAP subsidies for neither CF nor OF; Scenario 3: grain prices for CF and OF were estimated as the average price between years 2013 and 2014; Scenario 4: wheat grain prices for CF were estimated as the average price between August and September (the common harvest months); Scenarios 5 and 6: wheat grain prices for CF were estimated as the January and December prices (respectively) for the particular cropping season; and Scenario 7, no cost for organic fertilizer. For blank cells, the values correspond to the baseline scenario.

## Supplementary Table 4: Number of farmers who maximize gross margins and/or yield with their current practices

|  | CF | |  | OF | |  | Total | |
| --- | --- | --- | --- | --- | --- | --- | --- | --- |
|  | N Farmer | %Fields |  | N Farmer | %Fields |  | N Farmer | %Fields |
| Total | 14 |  |  | 9 |  |  | 23 |  |
| GM and yield are maximized | 6 | 28% |  | 3 | 15% |  | 9 | 23% |
| Only Yield is maximized | 2 | 6% |  | 5 | 33% |  | 7 | 17% |
| Only GM is maximized | 3 | 6% |  | 2 | 33% |  | 5 | 17% |

The Table shows the number of conventional (CF) and organic farmers (OF) (N Farmer) who maximized their gross margins (GM) and/or yield with their current practices, i.e. none of the treatments applied to their fields provided a better GM and/or yield. The “% of Fields” shows the average percentage of fields per farmer in which the maximum value (gross margin or yield) was achieved. For example, if farmer X had three fields in our experiment, and maximum yield was achieved in one field, its “%Fields“=33%; the values per farmer were averaged.

## Supplementary Table 5: Comparison between experimental controls and highest gross margins plots.

|  | CF | | |  | OF | | |
| --- | --- | --- | --- | --- | --- | --- | --- |
|  | Control | Best treatment | ∆ |  | Control | Best treatment | ∆ |
| Yield (t ha^-1^) | 7.5 (±2.3) | 7.8 (±2.7) | +4.0% |  | 2.9 (±1.6) | 3 (±1.7) | +3.4% |
| Total Revenue (€ ha^-1^) | 1838.3 (±488.9) | 1887.7 (±552) | +2.7% |  | 1366.2 (±624.3) | 1410.6 (±645.3) | +3.2% |
| Gross Margin (€ ha^-1^) | 1087.5 (±479.2) | 1288.5 ( 598.8) | +18.5% |  | 912.1 (±574.9) | 1122.7 (±568.1) | +23.1% |
| N input (kg ha^-1^) | 160.9 (±40.7) | 126.7 (±77.8) | -21.3% |  | 18.5 (±20.1) | 5.2 (±13) | -71.9% |
| N costs (€ ha^-1^) | 249.5 (±76.6) | 189.9 (±117.6) | -23.9% |  | 94.5 (±88.8) | 21.5 (±60.9) | -77.2% |
| Weed control intensity (WCI ha^-1^) | 1.5 (±0.8) | 0.8 (±0.8) | -46.7% |  | 1.9 (±1.8) | 0.9 (±1.6) | -52.6% |
| Weed control costs (€ ha^-1^) | 105.3 (±43.2) | 61.4 (±56.2) | -41.7% |  | 32.5 (±33.3) | 15 (±29.2) | -53.8% |
| Total Variable costs (€ ha^-1^) | 750.8 (±193.3) | 629.7 (±204) | -16.1% |  | 430.9 (±205.8) | 268.3 (±140.1) | -37.7% |

The Table shows, for both conventional (CF, N=33) and organic farming (OF, N=22), the means (±SD) for several parameters for experimental controls and respective plots in which the highest gross margins was achieved (i.e best economic strategy per plot). Positive values in ∆ columns indicate an increase, negative values a decrease, in comparison with control plots. The columns “best treatment” also includes plots where gross margins were maximized with the farmers’ current practices.

## Supplementary Table 6: Experimental Nitrogen and weed control inputs for 2012/2013 (a) and 2013/2014 (b)

(a)

| Treatment | Treatment abbreviation | Farming system | Design | Nitrogen inputs (mean ±SD) | Weed control intensity  (mean ±SD) | Nitrogen cost (mean ±SD) | Weed control cost  (mean ±SD) |
| --- | --- | --- | --- | --- | --- | --- | --- |
| Control plot | WN | CF | Centre | 140.7 (±32.8) | 1 (±0.5) | 207.6 (±58.4) | 80.8 (±25.3) |
|  |  |  | Margin |  |  |  |  |
|  |  | OF | Centre | 15.6 (±20) | 1 (±1.4) | 58.1 (±75.9) | 15.3 (±21.6) |
|  |  |  | Margin |  |  |  |  |
| Normal weed control  No fertilisation | WN0 | CF | Centre | 140.7 (±32.8) | 1 (±0.5) | 207.6 (±58.4) | 80.8 (±25.3) |
|  |  |  | Margin |  |  |  |  |
|  |  | OF | Centre | 15.6 (±20) | 1 (±1.4) | 58.1 (±75.9) | 15.3 (±21.6) |
|  |  |  | Margin |  |  |  |  |
| No weed control  Normal fertilisation | W0N | CF | Centre | 140.7 (±32.8) | 1 (±0.5) | 207.6 (±58.4) | 80.8 (±25.3) |
|  |  |  | Margin |  |  |  |  |
|  |  | OF | Centre | 15.6 (±20) | 1 (±1.4) | 58.1 (±75.9) | 15.3 (±21.6) |
|  |  |  | Margin |  |  |  |  |
| No Weed control  No Fertilisation | W0N0 | CF | Centre | 140.7 (±32.8) | 1 (±0.5) | 207.6 (±58.4) | 80.8 (±25.3) |
|  |  |  | Margin |  |  |  |  |
|  |  | OF | Centre | 15.6 (±20) | 1 (±1.4) | 58.1 (±75.9) | 15.3 (±21.6) |
|  |  |  | Margin |  |  |  |  |

| Treatment | Treatment abbreviation | Farming system | Nitrogen inputs (mean ±SD) | Weed control intensity (mean ±SD) | Nitrogen cost (mean ±SD) | Weed control cost (mean ±SD) |
| --- | --- | --- | --- | --- | --- | --- |
| Control plot | WN | CF | 171.1 (±41.1) | 1.7 (±0.8) | 270.4 (±77.2) | 117.5 (±45.5) |
|  |  | OF | 20.6 (±19.6) | 2.1 (±1.9) | 105.5 (±88.3) | 36.3 (±34.7) |
| No weed control  No fertilisation | W0N0 | CF | 0.0 (±0.0) | 0.0 (±0.0) | 0.0 (±0.0) | 0.0 (±0.0) |
|  |  | OF | 0.0 (±0.0) | 0.0 (±0.0) | 0.0 (±0.0) | 0.0 (±0.0) |
| No weed control  Lower fertilisation | W0N- | CF | 111.3 (±43.5) | 0.0 (±0.0) | 205.6 (±90) | 0.0 (±0.0) |
|  |  | OF | 15.8 (±16.3) | 0.0 (±0.0) | 122.7 (±13.9) | 0.0 (±0.0) |
| No weed control  Normal fertilisation | W0N | CF | 164.2 (±39.9) | 0.0 (±0.0) | 263.8 (±76.9) | 0.0 (±0.0) |
|  |  | OF | 26.2 (±15.5) | 0.0 (±0.0) | 118.1 (±76.6) | 0.0 (±0.0) |
| No weed control  Higher fertilisation | W0N+ | CF | 217.4 (±37.7) | 0.0 (±0.0) | 328.3 (±69.8) | 0.0 (±0.0) |
|  |  | OF | 35.5 (±18) | 0.0 (±0.0) | 136.4 (±99) | 0.0 (±0.0) |
| Lower weed control  No fertilisation | W-N0 | CF | 0.0 (±0.0) | 1 (±0.5) | 0.0 (±0.0) | 76.7 (±37.1) |
|  |  | OF | - | - | - | - |
| Lower weed control  Lower fertilisation | W-N- | CF | 111.3 (±43.5) | 1 (±0.5) | 205.6 (±90) | 76.7 (±37.1) |
|  |  | OF | - | - | - | - |
| Lower weed control  Normal fertilisation | W-N | CF | 158.9 (±45.1) | 0.9 (±0.5) | 264.4 (±88.9) | 69.2 (±28.9) |
|  |  | OF | - | - | - | - |
| Normal weed control  No fertilisation | WN0 | CF | 0.0 (±0.0) | 1.7 (±0.9) | 0.0 (±0.0) | 112.7 (±43.8) |
|  |  | OF | 0.0 (±0.0) | 2.3 (±1) | 0.0 (±0.0) | 38.8 (±19.8) |
| Normal weed control  Higher fertilisation | WN+ | CF | 217.5 (±40.2) | 1.8 (±0.9) | 334.3 (±72.3) | 120.3 (±43.8) |
|  |  | OF | 36.9 (±18.2) | 2.4 (±2.2) | 147.7 (±96.1) | 40.7 (±39.8) |
| Normal weed control & hand weeding  No fertilisation | W+N0 | CF | 0.0 (±0.0) | 1.6 (±0.8) | 0.0 (±0.0) | 115.9 (±40.4) |
|  |  | OF | 0.0 (±0.0) | 2 (±1) | 0.0 (±0.0) | 33.4 (±19.3) |
| Normal weed control & hand weeding  Lower fertilisation | W+N- | CF | 111.3 (±43.5) | 1.7 (±0.8) | 205.6 (±90) | 122.4 (±48.8) |
|  |  | OF | 15.8 (±16.3) | 1.6 (±0.9) | 122.7 (±13.9) | 27 (±18.7) |
| Normal weed control & hand weeding  Normal fertilisation | W+N | CF | 165.7 (±36.2) | 1.7 (±0.9) | 257.8 (±66.3) | 112.7 (±43.8) |
|  |  | OF | 20.5 (±14.8) | 2.3 (±2.2) | 86.5 (±79.9) | 39.9 (±39.2) |

Tables show, for both conventional (CF) and organic farming (OF) involved in the experiment, the means (±SD) input values and costs in terms of nitrogen (N) and weed control (weed control intensity, WCI). Control plots represent the actual farmer management strategy. WCI, for CF this is TFI (treatment frequency index) whereas for OF this is the number of times the field was mechanically weeded). The letters W and N with the suffixes “0”, “-” or “+” indicate no, reduced or higher fertilization or weed control; suffix “h” indicates normal weed control plus hand weeding, and WN is the control treatment. Due to the differences in the experimental design, normal and “zero” doses include plots for both cropping seasons (2012/2013 and 2013/2014) whereas the other treatments are for the 2013/2014 cropping season only. There were no reduced weed control treatments for OF.

## Supplementary Table 7: References for crop prices

| Winter cereal variety | Grain prices (€.T^-1^) | | | | |
| --- | --- | --- | --- | --- | --- |
|  | CF | |  | OF | |
|  | 2013 | 2014 |  | 2013 | 2014 |
| Winter wheat | 215 | 187 |  | 363 | 367 |
| Barley | 192 | 155 |  | 395 | 395 |
| Spelt | - | - |  | 390 | 390 |
| Triticale | - | - |  | 289 | 289 |

Grain prices for 2013 and 2014 were set equal to the average annual market values for CF^1,2^ and the average cooperative price for OF^3–5^.

## Supplementary Table 8: References for c**ost of inputs (fertilizers, regulators, herbicides and machinery)**

| Description | References |
| --- | --- |
| Nutrients | 6–9 |
| Nutrient prices | 10,11 |
| Plant growth regulator prices | 12–18 |
| Herbicide prices | 16,19–24 |
| Fungicide prices | 17,18,25–36 |
| Molluscicide prices | 18,37 |
| Insecticide prices | 18 |
| Seed prices | 38 |
| Machinery prices | 39 |

The Table shows the different input groups and references for determining costs as well as the nutrient composition of fertilizers (Nitrogen, N, Phosphorus, P, Potassium, K, and Sulphate, S): i) For fertilizers that were not found in the references, the composition was determined by personal communication with an expert. The same nutrient prices were used for the calculation of fertilizer cost for both conventional (CF) and organic farming (OF) as it was assumed that inorganic and organic nutrients had the same price; ii) Plant growth regulator prices. Plant growth regulators (hormones) may be either biostimulants or bioinhibitors and are compounds, other than nutrients, that can modify plant physiological processes; iii) Herbicide prices. Herbicides are the main pesticides used by farmers in both quantity and cost. Prices do not fluctuate greatly vary between years (personal communication), but they vary significantly between products (in our study lowest cost was 5.5 €.L^-1^ whereas the highest was 775 €.L^-1^) being strongly dependent on the active ingredients and concentrations; iv) Fungicide prices. Fungicides are used to prevent the spread of fungi on the plants; v) Molluscicide prices. Molluscicides are used against molluscs, such as snails; vi) Insecticide prices. In our sample only one insecticide product was used; vii) Seed prices. Based on information from the cooperatives, the variations in seed cost per hectare are very small and almost negligible within the total activity costs. The price used was the average certified seed price, fungicide treated and packaged. Seeds coming directly from the farm itself are assumed to cost 50% of their certified counterpart, and certified organic seeds to cost 50% more; and viii) Machinery prices. The price per hectare of machines used takes in consideration the average price of hiring from a local cooperative.

# Supplementary figures

## Supplementary Figure 1: Experimental design in 2012/13 (A) and in 2013/14 (B) cropping seasons.

A) In 2012-2013 cropping seasons, five experimental plots were set up in the field: two in the field margin (here defined as the five first border in the field) and three in the field core. The five plots were divided in two sub-plots and one of the sub-plot did not received any nitrogen (N0) supply. Weed control was stopped (W0) in one of the two plots in the field margin. B) In 2013-2014 cropping season, the experimental area was placed in the field core and was divided in four sub-plots corresponding to the four treatment: normal N supply and weed control (NW), No N supply and weed control (N0W), N supply and no weed control (NW0) and no N supply and no weed control (N0W0). Red squares indicate the areas in which hand weeding was performed (W+ treatment). Reduced N (N-) and weed control (W-) treatments were applied at the experimental plot scale in half of the fields (Fig. B1). In these fields, the farmers reduced both N supply and weed control in the experimental area. Increased N treatment (N+) was only applied in the other half field (i.e. with current farmers’ practices in the experimental plot; blue square in Fig. B2). In all fields, crop were harvested out of the experimental plot (extra plot). Consequently, the treatments were distributed as follows: in all fields in the 2012-2013 cropping season: N0W0, N0W, NW0 and NW; in half of the fields in the 2013-2014, NW, N0W, N0W+, N-W+, NW+, N-W-, N0W-, N0W0, N-W0 and in the other half, N0W0, N0W+,NW0, NW+, NW, N+W, N0W, N0W+.

A.

B.


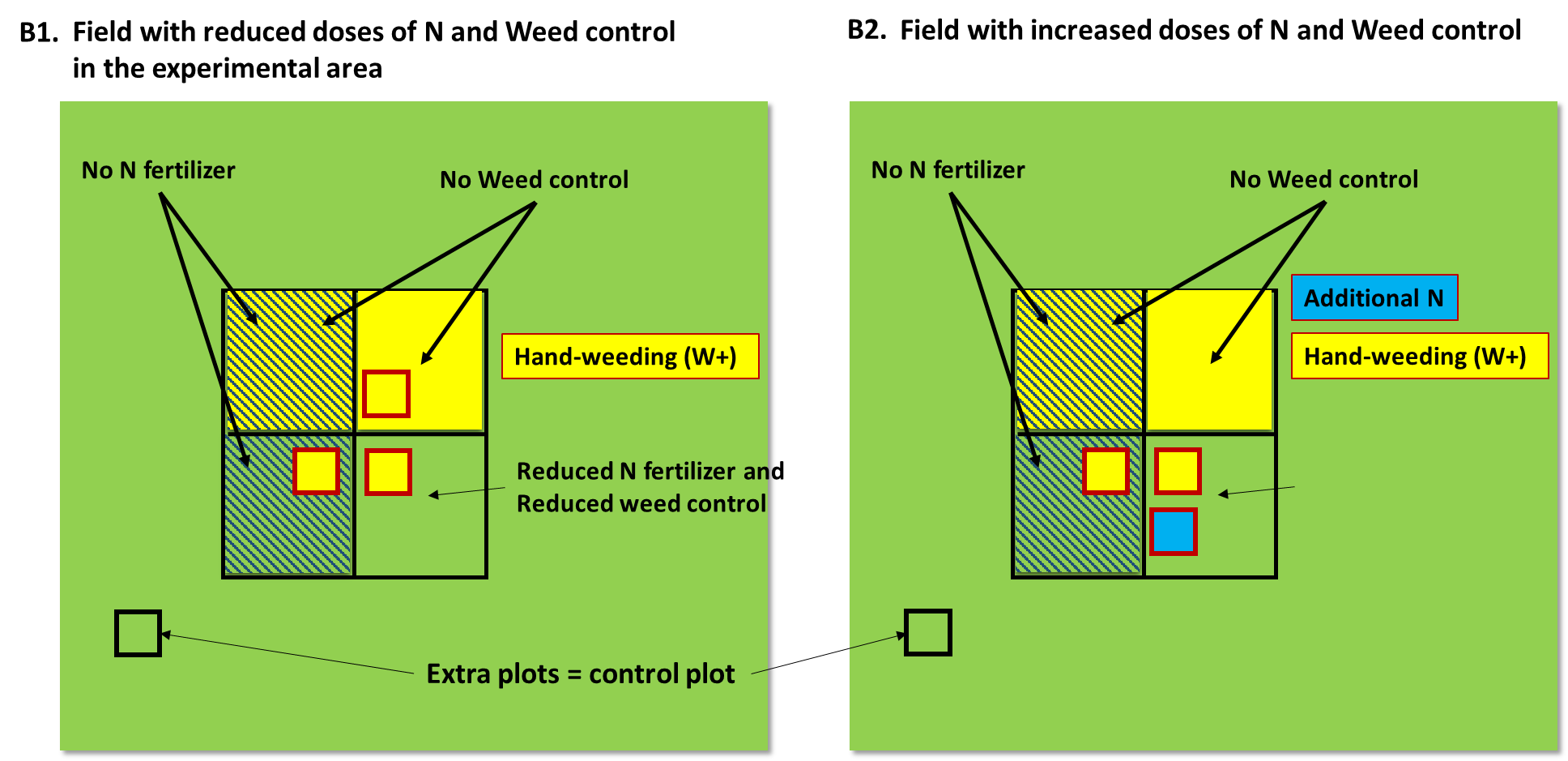


## Supplementary Figure 2: Robustness test under seven different economic scenarios.

The upper figures indicate the outcomes for the sensitivity of GM to prices using seven scenarios (b-h) in which CAP subsidies, grain prices or fertilizer prices were modified as in Supplementary Table 3. Solid and dashed lines represent significant and non-significant relationships, respectively. The blue line represents the response for conventional farmers and the green line for organic farmers. Figures a) baseline scenario; b) Scenario 1: inclusion of CAP pillar 2 (agro-environmental measures subsidies); c) Scenario 2: no CAP subsidies for neither CF nor OF; d) Scenario 3: grain prices for CF and OF were estimated as the average price between years 2013 and 2014; e) Scenario 4: wheat grain prices for CF were estimated as the average price between August and September (the common harvest months); f and g) Scenarios 5 and 6: wheat grain prices for CF were estimated as the January and December prices (respectively) for the particular cropping season; and h) Scenario 7, no cost for organic fertilizer. See Supplementary Table 3 for further details on used prices. Similar trends are observed for six of the seven scenarios (Fig. 1), i.e. GM decreases significantly with the costs of weed control and N fertilization in CF, but no significant relationship was found for OF fields. We detected, however, that when CAP subsidies are not included (plot c), 15% (5) and 10% (2) of CF and OF farmers, respectively, had negative gross margins. Therefore, changing the prices of either inputs or the output have no influence on the general results


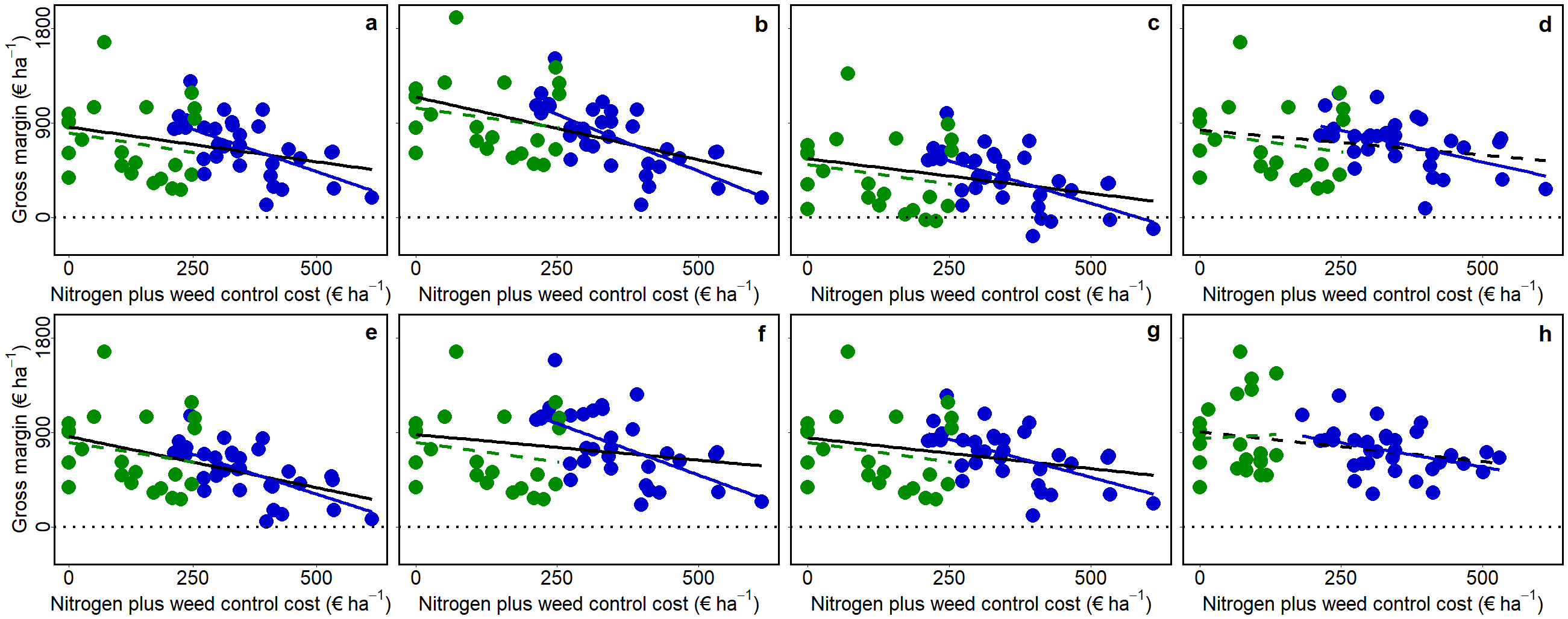


## S****upplementary Figure 3: Relationship between N input and weed control in terms of quantity applied and costs.****

We investigated the relationship between N supply and weed control using data from farmers’ inquiries for conventional (CF) and organic farming (OF). For CF, we found a significant positive relationship between nitrogen (N) supply and herbicide intensity (TFI) (Pearson correlation test: r=0.392, p-value<0.001). This suggests that CF farmers applied a more intense weed control when N supply is high. Consequently, N and weed control costs were also significantly positively related (r=0.386, p-value<0.001). This relationship was not found in OF: weed control was not higher in fields with high N supply (r=0.19, p-value=0.094), hence N and weed control costs were not correlated (r=0.158, p-value= 0.1711).


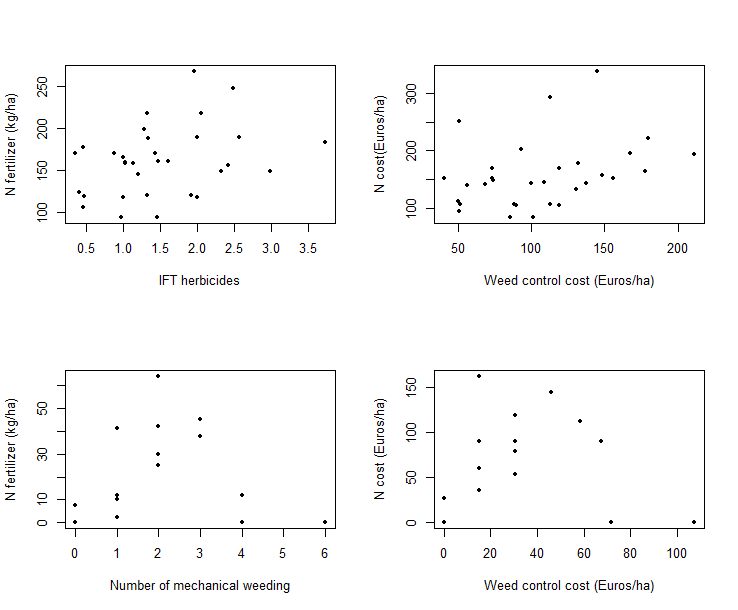


S**upplementary Figure 4: Relationship between the effect size ratio in Gross margin (A) and Yield (B), and farmers’ N supply in OF fields.**

## Effect size ratio were computed between control and (i) weed control reduction with no change in N supply (NW0/NW-) or (ii) weed control reduction when N supply is reduced (N-W0/N-W-). Treatments are indicated by the different colours: light green for N-W0/N-W- and dark green for NW0/NW-. Lines show linear relationships. Positive values indicate that GM (A) or Yield (B) are higher in treatment plots than in control ones. While negative values indicate that higher gross margins or yields are obtained under farmers’ current strategies (controls).


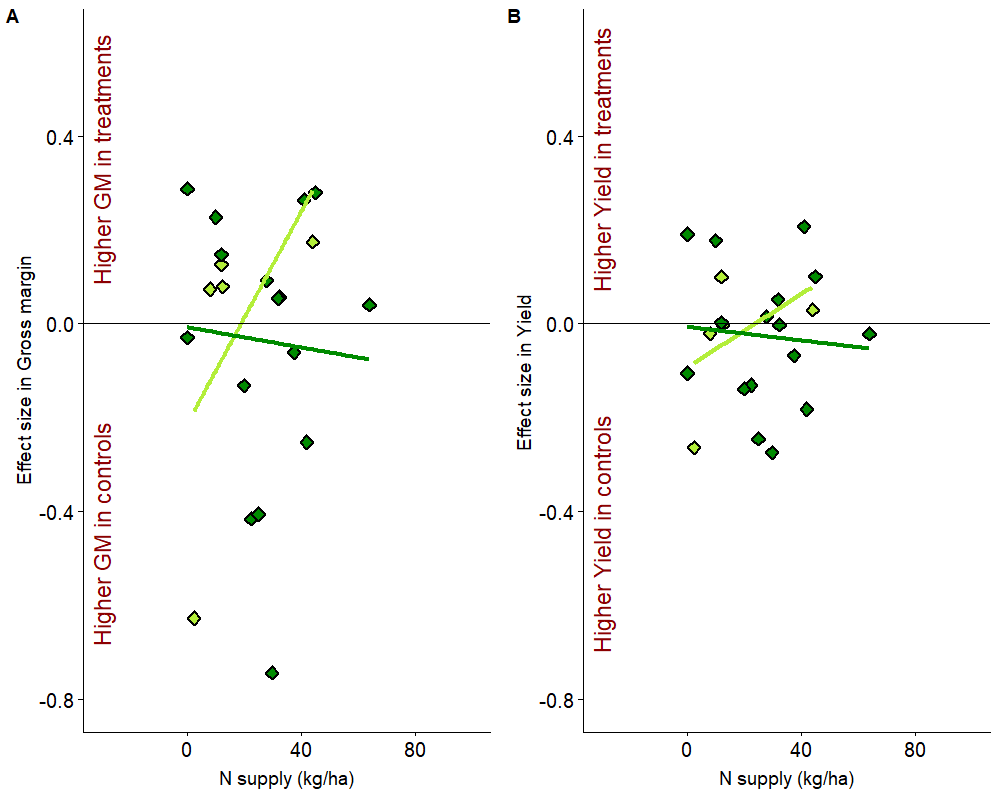


## Additional references in Supplementary Information

1. Finances.net. Matières premières - Cours temps réel , Blé. (2017). Available at: http://www.finances.net/matieres_premieres/historique/prix-ble/15.5.2010_15.6.2017. (Accessed: 1st March 2017)

2. FranceAgriMer. *Les prix payés aux producteurs - Résultats de l’enquête trimestrielle pour le 2e trimestre de la campagne 2013/14*. (2014).

3. FranceAgriMer. *Enquête annuelle prix à la production en agriculture biologique*. (2016).

4. Chambagri. *Résultats des essais céréales bio – Campagne 2012-2013*. (2013).

5. Chambagri. *Résultats des essais céréales bio – Campagne 2013-2014*. (2014).

6. Jeuffroy, M. & Recous, S. Azodyn: a simple model simulating the date of nitrogen deficiency for decision support in wheat fertilization. *Eur. J. Agron.* **10,** 129–144 (1999).

7. Acti-sol. Engrais naturel à usages multiples (pur fumier de poule) 5-3-2. 1 (2017). Available at: http://acti-sol.ca/engrais/engrais-naturel-a-usages-multiples-pur-fumier-de-poule-5-3-2/#onglets-produit. (Accessed: 15th February 2017)

8. COMIFER. *Calcul de la fertilisation azotee - Guide méthodologique pour l’établissement des prescriptions locales*. (2013).

9. FERTILEC. *Ferti-guide catalog*. (2007).

10. ARVALIS. Bilan des enquêtes 2014 - Chambres d’Agriculture de Poitou-Charentes. *Agricultures & Territoires - Chambres D’agriculture Poitou-Charentes* 1–20 (2015).

11. AGRILISA. Soufre. *Produit phytosanitaire prix* 1 (2017). Available at: http://www.agrilisa.com/Boutique/tabid/64/ProdID/40199/CatID/278/SOUFRE.aspx. (Accessed: 15th February 2017)

12. ARVALIS. *Varietes et traitments d’automne des cereales. Preconisations 2013-2014*. (2013).

13. ARVALIS. *Fertilisation des cereales*. (2013).

14. ARVALIS. *Guide Culture interventions de Printemps - Interventions de printemps, Campagne 2016-2017*. (Agricultures & Territoires - Chambre D’Agriculture Seine-et-Marne, 2016).

15. AGRILEADER. EpsoTop. 1 (2017). Available at: http://www.agrileader.fr/adjuvants/1882-0709500-epsotop.html. (Accessed: 15th February 2017)

16. ARVALIS. *Guide 2016 Protection des cultures*. (2015).

17. ARVALIS. Traitements et interventions de printemps - Preconisations 2012-2013. 1–185 (2012).

18. ARVALIS. in *Interventions de printemps 2016-2017* 30–144 (ARVALIS, 2016).

19. ARVALIS. in *Variétés et traitements d’automne* 133–188 (2012).

20. CAPLA. *Cultures D’Hiver - Intervetions du moment*. (2011).

21. CristalCoop. *Orientation agronomiques 2017*. (2016).

22. ARVALIS. *Agronomie et Desherbage des graminees en Cereales a Paille*. (2014).

23. ARVALIS. in *Variétés et traitements d’automne* 119–162 (ARVALIS, 2012).

24. Fourrages-Mieux. *Prix des herbicides*. (2011).

25. ARVALIS. *Cereales a paille - interventions de printemps*. (2016).

26. ARVALIS. Traitements et interventions de printemps - Preconisations 2013-2014. 1–180 (2013).

27. ARVALIS. *Semis Direct en Poitou-Charentes - Bilan des enquêtes 2013*. (ARVALIS, 2013).

28. ARVALIS. *Cereales a paille - interventions de printemps, Centre, Ile de France, Auvergne, Limosin*. (ARVALIS, 2016).

29. in *Lutte contre les maladies du ble tendre* (ed. ARVALIS) 85–129 (2012).

30. ARVALIS. in *Guide des Preconisations Cereales et Mais* 7–78 (Chambre d’agriculture de la Manche, 2016).

31. Agriclean. *Agriclean- Tarif 2015*. (2015).

32. ARVALIS. *Stratégies fongicides - Ble*. (2010).

33. ARVALIS. in *Interventions de printemps 2015-2016* 7–36 (2015).

34. CETIOM. *Guide de Culture - Colza*. (2014).

35. ARVALIS. *Maladies des feuilles et des épis – Fongicides – Blé tendre*. (2013).

36. ARVALIS. in *Guide des Preconisations Cereales et Mais* 36–78 (Chambre d’agriculture de la Manche, 2016).

37. ARVALIS. in *Choisir & decider - Synthese nationale* 233–256 (ARVALIS, 2014).

38. GNIS. *Avez-vous analysé tous les éléments liés au poste semence?* (2014).

39. ITAB. *Cas-types grandes cultures biologiques*. (2013).
